# The Impact of Wearable Technologies on Blood Pressure Control in Hypertensive Patients: A Systematic Review and Meta-Analysis

**DOI:** 10.7759/cureus.71220

**Published:** 2024-10-10

**Authors:** Mostafa Mohrag, Mohammed E Mojiri, Manar S Hakami, Mohammed S Alghamdi, Abdulaziz Y Moafa, Sawsan J Kreet, Saad M Alghamdi, Hams A Nasib, Omar R Alghamdi, Saleha M Ayoub, Aesha A Hakami, Sarah M Tawashi, Rola M Tayeb, Azezh A Kariry, Salem M Ayyashi

**Affiliations:** 1 Department of Medicine, Jazan University, Jazan, SAU; 2 College of Medicine, Jazan University, Jazan, SAU; 3 College of Medicine, Albaha University, Albaha, SAU; 4 College of Pharmacy, Jazan University, Jazan, SAU

**Keywords:** blood pressure control, cardiovascular disease, continuous monitoring, hypertension, lifestyle modifications, medication adherence, systematic review, wearable technology

## Abstract

Hypertension is a major global health concern and a leading risk factor for cardiovascular disease and premature death. Despite effective treatments, blood pressure control remains suboptimal due to challenges in medication adherence, lifestyle modifications, and regular monitoring. Wearable technologies offer potential solutions by providing continuous monitoring and promoting healthier behaviors. This systematic review and meta-analysis evaluated the impact of wearable technologies on blood pressure control in hypertensive patients. A comprehensive search of databases identified six randomized controlled trials, which varied in sample size, interventions, and outcomes. The meta-analysis revealed small, non-significant effects on systolic (standardized mean difference (SMD) = -0.27, 95%CI -0.57, 0.04) and diastolic (SMD = -0.16, 95%CI -0.41, 0.08) blood pressure, with considerable heterogeneity across studies. While wearable technologies show promise in hypertension management, their effects on blood pressure control are inconsistent. Future research should focus on more tailored interventions, identifying patient subgroups that may benefit most, and integrating wearables with traditional care to enhance hypertension management.

## Introduction and background

Hypertension, commonly known as high blood pressure, is a significant global health concern, affecting an estimated 1.28 billion adults worldwide [1]. It is one of the most critical risk factors for cardiovascular diseases, including heart attack, stroke, and heart failure, and is a leading cause of premature death globally. Despite its prevalence and the availability of effective treatments, hypertension remains poorly controlled in many patients, partly due to challenges related to medication adherence, lifestyle modifications, and the need for regular monitoring [2]. Given these challenges, there is growing interest in leveraging digital health technologies, particularly wearable devices, to support hypertension management and improve patient outcomes.

Wearable technologies such as smartwatches, fitness trackers, and connected blood pressure monitors have gained widespread popularity in recent years due to their ability to continuously monitor various physiological parameters, including heart rate, physical activity, sleep patterns, and blood pressure [3]. These devices provide real-time feedback to users, encouraging healthier behaviors and facilitating early detection of health issues. Moreover, when integrated with smartphone applications and telemedicine platforms, wearable technologies offer the potential for enhanced remote patient monitoring, personalized health interventions, and improved communication between patients and healthcare providers [4]. This integration is particularly appealing in managing chronic conditions like hypertension, where ongoing monitoring and timely adjustments to treatment plans are crucial.

The impact of wearable technologies on hypertension management has been the subject of numerous studies, but the results have been mixed. Some studies have shown that wearable devices can significantly improve blood pressure control, enhance medication adherence, and increase patient engagement in their own health management. For instance, studies by Kario et al. [5] and Contreras et al. [6] demonstrated that digital health interventions, including wearable technologies, can lead to meaningful reductions in systolic and diastolic blood pressure when combined with lifestyle modifications and regular monitoring. However, other studies, such as those by Morawski et al. [7] and Pletcher et al. [8], reported minimal or no significant differences in blood pressure outcomes between patients using wearable technologies and those receiving standard care. This variability in outcomes may be due to differences in study design, population characteristics, the types of devices used, and the duration of interventions.

Given the increasing adoption of wearable technologies in healthcare and the mixed evidence regarding their effectiveness in managing hypertension, there is a clear need for a systematic review to synthesize the existing evidence. This review aims to evaluate the impact of integrating wearable technologies on blood pressure control in hypertensive patients.

## Review

Methods

Literature Search Strategy

We adhered to the Preferred Reporting Items for Systematic Reviews and Meta-Analyses (PRISMA) guidelines during the conduct and reporting of this review. Our search strategy included four major online databases: PubMed, Web of Science (WOS), Scopus, and the Cochrane Central Register of Controlled Trials (CENTRAL), covering publications from inception until August 1, 2024. We employed specific keywords such as "smartphone applications," "wearable technology," "wearable devices," "smart wearables," "smartwatches," "hypertension," and "blood pressure." These keywords were combined using Boolean operators, and the search strategy was customized for each database. The search terms used were "smartphone applications" OR "wearable technology" OR "wearable devices" OR "smart wearables" OR "smartwatches" AND (hypertension OR "blood pressure"). Filters were applied to include only articles published in English, involving human participants, and randomized controlled trials. Additionally, we manually examined the reference lists of the included studies to identify any relevant articles that might have been missed during the initial search process.

Eligibility Criteria

We established the selection criteria using PIOCS (P-population, I-intervention, C-comparison, O-outcome, S-study design). We included only randomized clinical trials (RCTs) published in the English language that: included patients with poorly controlled blood pressure, used any technological application either alone or in combination with another interventional method, used usual care or standard treatment for comparison, and employed any outcome measure to assess the effect of the technological applications. We excluded observational studies, studies published in languages other than English, and published abstracts without full-text articles.

Study Selection

Two reviewers independently screened the titles and abstracts of the retrieved articles using predetermined eligibility criteria. Any disagreements or discrepancies were resolved by a third reviewer until a consensus was reached.

Data Extraction

The full texts of the included articles were further analyzed, and the following data were extracted: sample size, type of wearable technology used, age of participants, interventions in experimental and control groups, outcome measures, and main results. Any potential conflicts were resolved by a third reviewer.

Quality Appraisal

The methodological quality of the included studies was independently assessed by two reviewers using the modified Downs and Black Checklist for clinical trials. The checklist consists of 27 questions rating four categories: reporting, external validity, internal validity, and power. Studies are considered to be of excellent quality when the final score ranges from 26 to 28, good quality if the score ranges from 20 to 25, fair quality if the score ranges from 15 to 19, and poor if the score is 14 or less. Any disagreements or discrepancies were resolved by discussion until a consensus was reached.

Results

Study Selection

**Figure 1:**
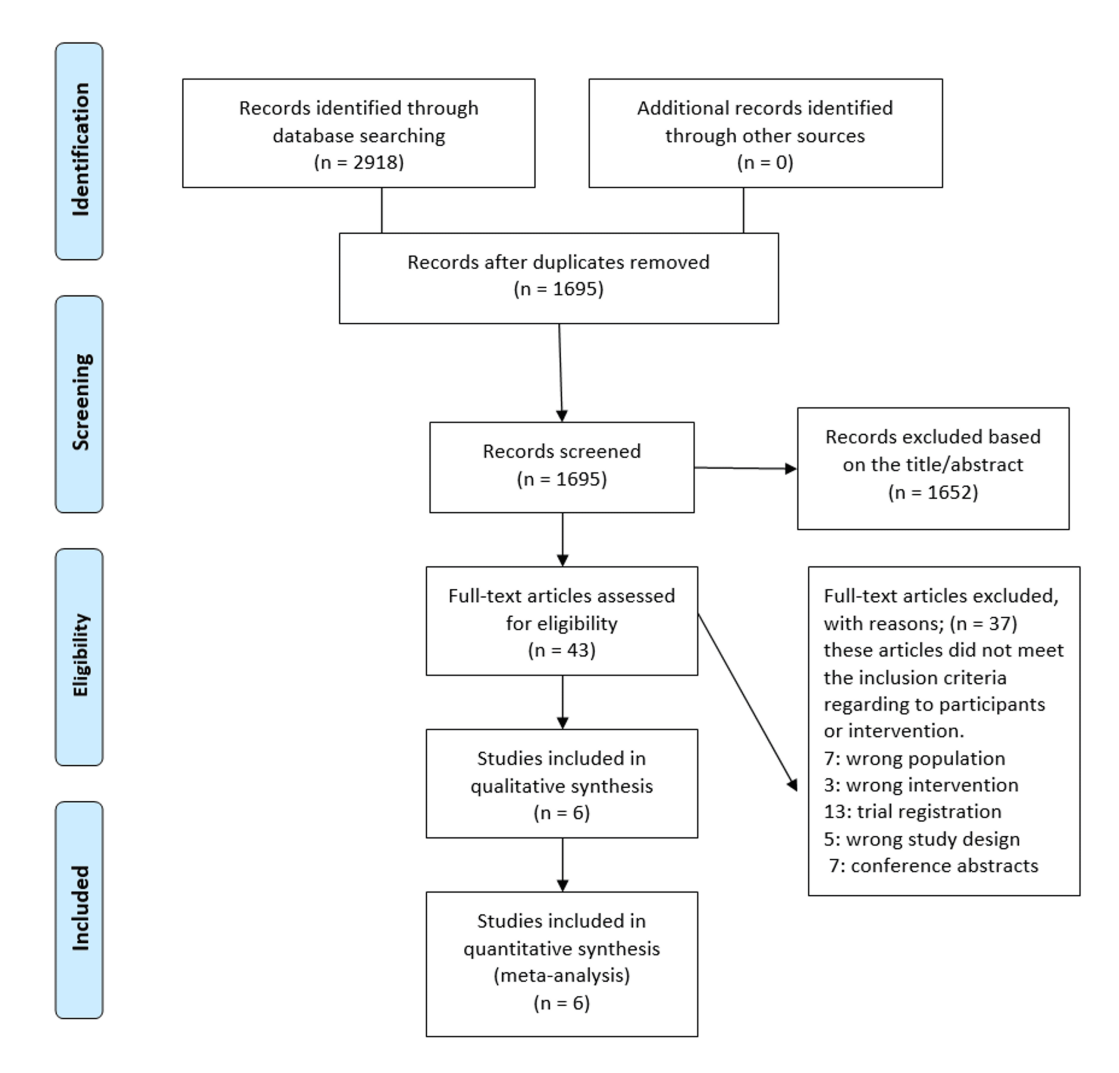
PRISMA flowchart of studies search and selection PRISMA: Preferred Reporting Items for Systematic Reviews and Meta-Analyses

The comprehensive search yielded a total of 2,918 papers: 376 from PubMed, 1,617 from Scopus, 620 from WOS, and 305 from CENTRAL. After removing 1,223 duplicate studies, 1,695 papers remained for screening. The titles and abstracts of these papers were screened, resulting in the exclusion of 1,652 studies, leaving 43 records for full-text screening. During the full-text screening phase, 37 articles were excluded for the following reasons: 14 were trial registrations, seven were conference abstracts, four involved the wrong intervention, five had the wrong study design, and seven involved the wrong population. Ultimately, six studies met the inclusion criteria and were included in the qualitative and quantitative analysis [5-10]. The PRISMA flowchart in Figure 1 illustrates the study selection process.

Characteristics of the Studies 

This systematic review includes six studies conducted in various countries, including Spain, the United States, and Japan, all RCTs, as shown in Table 1. Each study focuses on patients with different hypertension-related diagnoses, ranging from essential hypertension to poorly controlled hypertension. The sample sizes vary significantly, with the largest study involving 2,101 participants [8] and the smallest involving 16.

**Table 1:** Summary of studies evaluating wearable technology for hypertension management RCT: randomized clinical trial; BP: blood pressure; SBP: systolic blood pressure; EG: experimental group; CG: control group

Author, Year	Country	Design	Diagnosis	Sample Size	Age (years)	Type of Wearable Technology Used
Contreras et al., 2018 [6]	Spain	RCT	Patients with hypertension	154; EG (77), CG (77)	57.5 ± 9.9	AlerHTA app (Grupo 2-comunicacion Medica sl, Madrid, Spain) created for the Hypertensive Club of the Spanish Society of Hypertension; free and easily accessible. Allows users to record personal data, set BP goals, track doctor’s advice, schedule reminders, and log BP measurements.
Roberts et al., 2019 [10]	United States	RCT	Patients with moderate-to-high risk of coronary heart disease events	40; EG (20), CG (20)	EG: 72.1 (8.3); CG: 71.85 (6.5)	Hip-worn solid-state tri-axial accelerometer (ActiGraph GT3X; ActiGraph LLC, Pensacola, Florida, United States)
Kario et al., 2021 [5]	Japan	RCT	Patients with essential hypertension	390; EG (199), CG (191)	EG: 52.4 (8.1); CG: 52.0 (7.6)	Smartphone app (HERB Mobile system; CureApp Inc., Chuo-ku, Tokyo, Japan) connected to a home BP monitoring device
Baron et al., 2019 [9]	United States	RCT	Patients with short sleep duration and prehypertension/stage 1 hypertension	16; EG (11), CG (4)	45.8 ± 9.8	Sleep tracker (Fitbit Flex 2; Fitbit, Inc., San Francisco, California, United States)
Pletcher et al., 2022 [8]	United States	RCT	Patients with uncontrolled hypertension	2101; EG (1051), CG (1050)	58 ± 13	Blood pressure monitor paired with a smartphone application
Morawski et al., 2018 [7]	United States	RCT	Patients with poorly controlled hypertension	411; EG (209), CG (202)	EG: 51.7 (10.5); CG: 52.4 (10.1)	Bluetooth-enabled blood pressure monitor

The studies integrated various types of wearable technology to assist with hypertension management [5-10]. For instance, the AlerHTA app (Grupo 2-comunicacion Medica sl, Madrid, Spain) used in the Spanish study allows users to record personal data and track blood pressure (BP) goals. In the United States, technologies such as the ActiGraph GT3X (ActiGraph LLC, Pensacola, Florida, United States) accelerometer and Fitbit Flex 2 (Fitbit, Inc., San Francisco, California, United States) were utilized to monitor additional health metrics like physical activity and sleep patterns. The Japanese study by Kario et al. employed a smartphone app connected to a home BP monitoring device [5]. Participants' ages ranged from approximately 45 to 72 years, reflecting a focus on middle-aged and older adults. Each study aimed to explore how these wearable technologies could enhance hypertension management, with outcomes potentially including improved BP control and increased adherence to health recommendations. These findings contribute to understanding the role of wearable technology in managing chronic health conditions like hypertension (Table 2).

**Table 2:** Characteristics of included studies EG: experimental group; CG: control group; SMBP: self-measured blood pressure; SBP: systolic blood pressure; EX+NEPA: exercise + non-exercise physical activity; MMAS: Morisky Medication Adherence Scale; EX: exercise only

Author, Year	Intervention of EG	Intervention of CG	Period of intervention	Outcome measures	Results
Contreras et al., 2018 [6]	Patients used a smartphone app called AlerHTA (Grupo 2-comunicacion Medica sl, Madrid, Spain). The app promoted health education, reminders for appointments, and medication intake. Included instructions and support for app usage.	Received usual care for high blood pressure. Routine blood pressure monitoring every six months. Annual control of therapeutic adherence.	18 months in total: 6 months for inclusion and 12 months for follow-up.	Average adherence percentage (AP) Blood pressure control.	The study showed that using the AlerHTA smartphone app significantly improved medication adherence and blood pressure control among hypertensive patients. In the intervention group, daily adherence rates were notably higher, and blood pressure control improved to 38.6% compared to 17.8% in the control group. The absolute risk reduction was 23.64%, with a number needed to treat of 4.23. Overall, the app proved to be an effective tool for enhancing hypertensive patient management.
Roberts et al., 2019 [10]	EX+NEPA Participants received a wearable activity tracking device and behavioral monitoring.	EX; Participants underwent structured exercise without additional wearable technology	20 weeks total (8-week center-based exercise intervention followed by 12 weeks of continued activity).	Steps per day Sedentary time Blood pressure (systolic and diastolic) Exercise capacity (6-min walk test) Waist circumference Clinical metabolic profiles (cholesterol, triglycerides, glucose) Inflammatory and oxidative stress biomarkers	The study found that the EX+NEPA group, which used wearable technology, increased their daily steps by 1994 and reduced sedentary time by 6.8 minutes compared to the EX group. Systolic blood pressure decreased by 9.9 mmHg in the EX+NEPA group. While there were slight improvements in exercise capacity and waist circumference, the changes in metabolic and inflammatory markers were minimal. Overall, wearable technology positively influenced physical activity and some cardiovascular risk factors in older adults.
Kario et al., 2021 [5]	Digital therapeutics system (HERB system; CureApp Inc., Chuo-ku, Tokyo, Japan) + standard lifestyle modification.	Standard lifestyle modification alone.	12 weeks.	Mean change in 24-hour ambulatory SBP. Mean changes in office and home blood pressure.	The HERB-DH1 trial demonstrated that using a digital therapeutics system alongside standard lifestyle modifications significantly reduced 24-hour ambulatory, home, and office SBP compared to lifestyle modifications alone. The reductions were -2.4 mmHg for ambulatory SBP, -4.3 mmHg for morning home SBP, and -3.6 mmHg for office SBP. A greater percentage of the digital therapeutics group achieved target morning home blood pressure. Additionally, significant decreases in body weight and BMI were observed in the digital therapeutics group.
Baron et al., 2019 [9]	Technology-assisted sleep extension Included wearable sleep tracker, smartphone application, weekly lessons, and telephone coaching	Self-management Instructed to maintain current sleep schedule	6 weeks	Total sleep time, 24-hour systolic and diastolic blood pressure, Sleep disturbance, Sleep-related impairment	The study evaluated a six-week technology-assisted sleep extension intervention for adults with short sleep duration and prehypertension/stage 1 hypertension. The intervention group showed significant improvements in total sleep time, reduced 24-hour systolic and diastolic blood pressure, and better sleep quality compared to the control group. Participants in the intervention rated it highly and completed most of the coaching sessions. These findings suggest the intervention is effective and well-received for improving sleep and reducing blood pressure.
Pletcher, 2022 [8]	Participants were instructed to download and use the Medisafe app (Medisafe, Boston, Massachusetts, United States), which includes reminder alerts, adherence reports, and optional peer support.	Did not receive any intervention.	12 weeks	Change in self-reported medication adherence (measured by the MMAS) Change in systolic blood pressure Proportion of participants with well-controlled blood pressure (<140/90 mm Hg)	In a 12-week study, participants using the Medisafe app showed a small improvement in self-reported medication adherence compared to controls. The adherence improvement was 0.4 on the MMAS, with a statistically significant difference. However, there was no significant change in SBP between the groups. The app's use did not lead to better blood pressure control despite improved adherence.
Morawski, 2018 [7]	Enhanced SMBP using a device paired with a connected smartphone application	Standard SMBP using a standard device.	6 months	Reduction in SBP Patient satisfaction (Net Promoter Score)	In a randomized clinical trial, enhanced SMBP using a smartphone-connected device showed no significant advantage over standard SMBP in reducing SBP. Both groups experienced similar mean reductions: −10.8 mm Hg for the enhanced group and −10.6 mm Hg for the control group, with an adjusted difference of −0.19 mm Hg (P = .81). Blood pressure control to below 140/<90 mm Hg was slightly higher in the enhanced group (32%) compared to the control (29%). Patient satisfaction was comparable, with 70% of the enhanced group and 69% of the control group likely to recommend their device.

As shown in Table 3, Contreras et al. implemented an 18-month intervention using the AlerHTA app, leading to improved medication adherence and blood pressure control [6]. The experimental group had significantly higher adherence rates than the control group. Roberts et al. conducted a 20-week study with wearable technology to increase physical activity [10]. The EX+NEPA (exercise + non-exercise physical activity) group saw increased daily steps and reduced systolic blood pressure, demonstrating the benefits of combining exercise with technology.

**Table 3:** Impact of wearable technology on systolic and diastolic blood pressure in hypertensive patients SBP: systolic blood pressure; DBP: diastolic blood pressure; EG: experimental group; CG: control group

Author, year	Type of Wearable Technology Used	Pre-Intervention SBP Score	Post-Intervention SBP Score	Pre-Intervention DBP Score	Post-Intervention DBP Score
Contreras et al., 2018 [6]	Smartphone app (AlerHTA; (Grupo 2-comunicacion Medica sl, Madrid, Spain))	EG: 134.7 ± 14; CG: 134.47 ± 8	After 6 months: EG: 130.6 ± 12; CG: 134.47 ± 8 After 18 months: EG: 132.2 ± 12; CG: 134.4 ± 11	EG: 81.64 ± 8; CG: 81.9 ± 6.8	After 6 months: EG: 78.76 ± 8; CG: 82.2 ± 8 After 18 months: EG: 78.5 ± 7; CG: 81.4 ± 9
Roberts et al., 2019 [10]	ActiGraph GT3X (ActiGraph LLC, Pensacola, Florida, United States)	EG: 137.5; CG: 137.5	After 8 weeks: EG: 135.67 (3.33); CG: 140.11 (3.4) After 20 weeks: EG: 131.44 (3.47); CG: 141.33 (3.46)	EG: 74; CG: 74	After 8 weeks: EG: 74 (2.33); CG: 76 (1.4) After 20 weeks: EG: 72 (3.47); CG: 74 (1.46)

Kario et al. focused on a 12-week intervention using the HERB-DH1 digital therapeutics system [5]. This approach significantly reduced 24-hour ambulatory, home, and office systolic blood pressure when combined with lifestyle modifications, surpassing lifestyle changes alone. Baron et al. evaluated a six-week technology-assisted sleep extension intervention [9]. Participants showed improvements in sleep quality and reductions in systolic and diastolic blood pressure, indicating the effectiveness of sleep enhancement in managing hypertension. Pletcher et al. conducted a 12-week study using the Medisafe app (Medisafe, Boston, Massachusetts, United States), which resulted in a small improvement in self-reported medication adherence [8]. However, there was no significant change in systolic blood pressure compared to the control group. Morawski et al. explored a six-month intervention with a connected blood pressure device [7]. The study found no significant advantage in reducing systolic blood pressure compared to the standard self-measured blood pressure group, although both groups showed similar reductions.

Overall, these studies highlight the varied applications of technology, with intervention periods ranging from six weeks to 18 months. Despite different focuses such as medication adherence, physical activity, digital therapeutics, and sleep quality, all demonstrate potential in improving hypertension management.

Quality Assessment

As shown in Table 4, Contreras et al. achieved high scores in reporting due to clear descriptions of hypotheses, patient characteristics, and interventions [6]. However, their study lacked blinding, affecting its internal validity for bias. Despite this, the study effectively adjusted for follow-up times and utilized appropriate statistical tests, ensuring reliability in its findings. Roberts et al. also excelled in reporting, with comprehensive details on outcomes and variability [10]. The study faced challenges in external validity due to the limited representation of the broader population. It maintained internal validity by using reliable compliance measures and accurate outcome assessments, though it did not employ blinding procedures.

**Table 4:** Summary of quality scores for selected hypertension research studies The methodological quality of the included studies was independently assessed by two reviewers using the modified Downs and Black Checklist for clinical trials.

Category	Contreras et al., 2018 [6]	Roberts et al., 2019 [10]	Kario et al., 2021 [5]	Baron et al., 2019 [9]	Pletcher et al., 2022 [8]	Morawski et al., 2018 [7]
Reporting	8	9	10	8	8	8
External Validity	3	3	2	0	1	3
Internal Validity - Bias	6	5	5	4	5	5
Internal Validity - Confounding	3	3	6	4	4	5
Power	1	0	1	0	1	1
Total Score	21					

The study by Kario et al. stood out with strong scores across most criteria [5]. The study clearly outlined its objectives and balanced confounders, contributing to its robust internal validity. While it was an open-label study, it still managed to adjust for potential confounding factors effectively. However, its external validity was restricted by the specific demographic studied. Studies by Baron et al. [9] and Pletcher et al. [8], varied in performance, while Morawski et al.'s study [7] highlighted the need for sufficient statistical power and thorough methodology to ensure significant clinical outcomes in managing hypertension.

Effect of Intervention on Medication Adherence

In the study by Contreras et al. [6], significant differences were observed between the intervention group (IG) and control group (CG) in adherence measures. Over six and 12 months, the IG showed higher adherence rates across various parameters. The percentage of adherents taking a single daily dose was notably higher in the IG, with 93.15% and 86.3% at six and 12 months, respectively, compared to 70.66% and 62.66% in the CG. The IG also demonstrated superior global adherence and correct time adherence, with percentages significantly favoring the intervention. These results underscore the effectiveness of the intervention in improving medication adherence among participants.

Pletcher et al. showed that after 12 weeks of follow-up, the mean (SD) adherence rose by 0.4 (1.5) in the IG, while it stayed the same in the CG, showing a between-group difference of 0.4 (95%CI, 0.1-0.7; P = .01) [8]. These findings remained consistent even after adjusting for baseline differences in secondary analyses and complete case analyses.

Effect of Intervention on Systolic Blood Pressure

**Figure 2:**
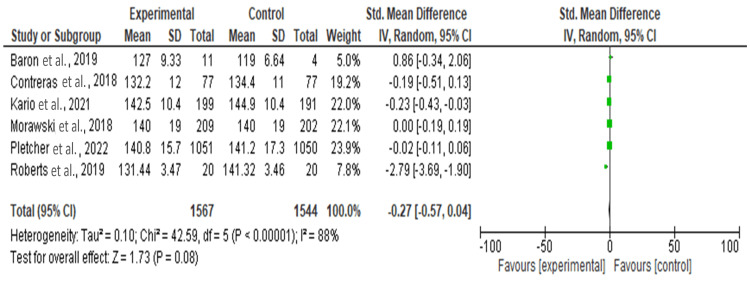
Effect of intervention on systolic blood pressure References: Baron et al. [9], Contreras et al. [6], Kario et al. [5], Morawski et al. [7], Pletcher et al. [8], Roberts et al. [10]

Figure 2 shows the forest plot that summarizes the results of six studies comparing experimental and control groups. Each study is represented by a line and a square, where the square's size reflects the study's weight in the analysis. The x-axis shows the standardized mean difference (SMD) with a 95% CI. For instance, the study by Roberts et al. [10] shows a significant negative effect favoring the CG, as its CI does not cross zero. Meanwhile, other studies by Pletcher et al. [8] and Morawski et al. [7], have CIs that cross zero, indicating no significant difference between groups.

At the bottom, the overall effect was represented by a diamond, summarizing the combined SMD across all studies. The overall SMD is -0.27 with a 95%CI of -0.57, 0.04, suggesting a slight, non-significant trend favoring the control group. The test for overall effect yields a P-value of 0.08, which is not statistically significant. The high heterogeneity, indicated by an I² of 88%, suggests substantial variability among the studies, meaning the results should be interpreted with caution.

After conducting the sensitivity analysis, the meta-analysis examined the effect of the intervention across five studies. The study by Roberts et al. [10] was identified as an outlier due to its extreme negative effect size, contributing to the heterogeneity. The overall effect size was small and non-significant (SMD = -0.07, 95%CI -0.20, 0.05), with moderate heterogeneity (I² = 40%). Removing Roberts et al.'s study in a sensitivity analysis would likely reduce heterogeneity but would not substantially change the overall conclusion, which indicates no significant difference between the experimental and control groups.

Effect of Intervention on Diastolic Blood Pressure

**Figure 3:**
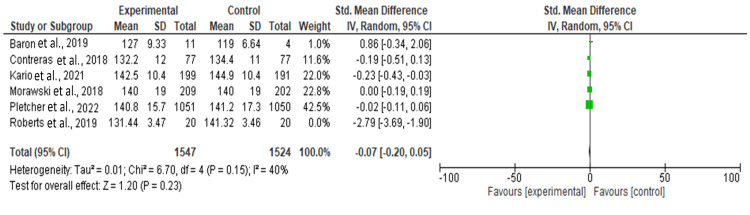
Effect of intervention on diastolic blood pressure References: Baron et al. [9], Contreras et al. [6], Kario  et al. [5], Morawski et al. [7], Pletcher et al. [8], Roberts et al. [10]

Figure 3 shows the forest plot that summarizes the results of five individual studies comparing an experimental group to a control group, with the effect size measured as SMD and 95%CI. The studies vary in their contribution to the overall analysis, with weights ranging from 3.9% [9] to 31.2% [7]. The individual SMDs range from -0.74 [10] to 0.08 [5], indicating varied effects across the studies. Most CIs cross zero, suggesting that the individual studies do not show statistically significant differences between the experimental groups and the CGs.

The overall pooled effect size across all studies was -0.16 (95%CI -0.41, 0.08), which is not statistically significant (Z = 1.29, P = 0.20). The heterogeneity among the studies was moderate to substantial, with an I² value of 62%, indicating that some of the variability in effect sizes could be due to differences between the studies rather than chance alone. The Chi² test for heterogeneity was significant (P = 0.03), further supporting the presence of heterogeneity. This suggests that while the overall effect is not significant, the differences in the effects observed across studies warrant further exploration.

## Conclusions

While digital health technologies hold promises for improving hypertension management, the evidence remains mixed, with significant variability in outcomes across different studies. This meta-analysis underscores the need for more tailored, multi-component interventions and emphasizes the importance of patient engagement and sustained use. Future research should focus on standardizing interventions and exploring the factors that contribute to their success, ensuring that these technologies effectively complement traditional hypertension management strategies.
